# SEROUS CYSTADENOMA OF PANCREAS: WHY THERE IS LOW ACCURACY IN IMAGING EXAMS?

**DOI:** 10.1590/0102-672020210002e1640

**Published:** 2022-01-31

**Authors:** Enio Campos AMICO, Caio Trajano Siqueira SALGADO, Luisa Maciel EMERENCIANO, Guilherme Augusto Santos FERREIRA, Jose Roberto ALVES, Luiz Eduardo Oliveira Forte Ferreira de SOUZA, José Sandro Pereira da SILVA

**Affiliations:** 1 Centro de Gastroenterologia e Endoscopia Digestiva de Natal, Hospital Universitário Onofre Lopes da Universidade Federal do Rio Grande do Norte (HUOL - UFRN) - Natal, Rio Grande do Norte (RN), Brasil.

**Keywords:** Cystadenoma, Serous, Pancreatic Neoplasms, Pancreatic Cyst, Cistoadenoma Seroso, Neoplasias Pancreáticas, Cisto Pancreático

## Abstract

Many patients with serous cystadenoma of the pancreas (SCP) underwent surgery due to diagnostic doubt.

**AIM:**

The aim of this study was to analyze the causes of low accuracy in diagnosing SCP.

**METHODS:**

This is a retrospective study of patients with SCP from a database of two hepatopancreatic biliary surgery outpatient clinics between 2006 and 2020. Patients with typical SCP lesions in imaging exams (e.g., tomography, magnetic resonance imaging [MRI], and endoscopic ultrasound [EUS]) and patients whose pathological testing confirmed this diagnosis were included.

**RESULTS:**

A total of 27 patients were included in this study. Most patients were women (85.18%), and the mean age was 63.4 years. Only one patient had typical pancreatitis symptoms. MRI was the most performed method (62.9%). The lesion was single in 88.9%, and the average size was 4 cm. The typical microcystic aspect was found in 66.6%. EUS was performed in 29.6% of cases. The mean carcinoembryonic antigen value in patients undergoing cyst puncture was 198.25 ng/mL. Surgical treatment was performed in 10 cases (37%). The cause of surgery in seven of these cases was due to a suspicion of mucinous cystadenoma based on an identification of atypical lesions (unilocular with or without septa and macrocystic) in imaging exams. A suspicion of intraductal papillary mucinous neoplasm with “worrying factors” was the indication for surgery in two cases. The last case underwent surgical treatment for a solid-looking lesion which was suspected of cancer. The complication rate ≥Clavien-Dindo 2 was 30%, and the clinically relevant pancreatic fistula rate (B and C) was 30%. Mortality was nil.

**CONCLUSION:**

The atypical morphological presentation of SCP, particularly unilocular and macrocystic lesions, is the main indication for surgery. Only the implementation of new, efficient, and reproducible diagnostic methods can reduce the number of unnecessary surgeries among these patients.

## RESUMO - RACIONAL

Apesar da recomendação atual que o cistoadenoma seroso deva ser tratado de forma conservadora, significativa parte dos pacientes com essa condição ainda é operada por dúvida diagnóstica.

**OBJETIVO:**

Analisar causas da baixa acurácia diagnóstica do cistoadenoma seroso.

**MÉTODOS:**

Estudo retrospectivo de portadores de cistoadenoma seroso de um banco de dados de dois ambulatórios de cirurgia hepatopancreaticobiliar entre 2006 e 2020. Foram incluídos pacientes com lesões típicas de cistoadenoma seroso aos exames de imagem (tomografia computadorizada, ressonância magnética e ecoendoscopia) e pacientes que o anatomopatológico confirmasse esse diagnóstico.

**RESULTADOS:**

27 pacientes foram incluídos. 85,18% eram do sexo feminino. A idade média foi de 63,4 anos. Apenas um apresentava sintomas típicos de pancreatite. A Ressonância magnética foi o exame mais realizado (62,9%). A lesão era única em 88,9% e o tamanho médio foi 4 cm. O aspecto típico microcístico foi encontrado em 66,6% dos casos, os demais foram considerados atípicos. A ecoendoscopia foi realizada em 29,6%. O valor médio de antígeno carcinoembrionário nos pacientes submetidos à punção do cisto foi de 198,25 ng/mL. O tratamento cirúrgico foi realizado em 10 casos (37%). Em 7, a causa cirúrgica foi a suspeita do cistoadenoma mucinoso mediante identificação de lesões atípicas (unilocular com ou sem septos e macrocística). Em 2, a suspeita de neoplasia papilar intraductal mucinosa com “fatores preocupantes” foi a indicação cirúrgica. O último foi submetido à cirurgia por lesão de aspecto sólido e suspeita de câncer. O índice de complicações > ou = Clavien-Dindo 2 foi 30%, o índice de fístula pancreática clinicamente relevante (B e C) foi 30%. A mortalidade foi nula.

**CONCLUSÃO:**

A apresentação morfológica atípica do cistoadenoma seroso, particularmente lesões uniloculares e macrocísticas, é a principal responsável pela indicação cirúrgica. Apenas a implementação de novos, eficientes e reprodutíveis métodos diagnósticos poderá reduzir o número de cirurgias desnecessárias nesses pacientes.

## INTRODUCTION

Serous cystadenoma of the pancreas (SCP) is a type of pancreatic cystic neoplasm (PCN) frequently found in clinical practice due to the growing number of imaging tests performed today. With a good clinical course and negligible risk of malignancy, it is currently recognized that conservative treatment is adequate for the vast majority of patients. Despite this, a significant number of patients with SCP are still operated on, and according to most studies, this is due to the low accuracy of imaging exams^13^. For example, in a recent study^23^, only one in four of the 133 patients with SCP who were resected were correctly diagnosed before surgery.

The reasons for this low accuracy remain unclear. Using a series of patients with SCP, the aim of the present study was to (1) analyze the accuracy of imaging/endoscopic exams in diagnosing SCP and (2) evaluate the causes of diagnostic errors in patients with SCP.

## METHODS

Patients from a database of the first author (ECA) treated between 2006 and 2020 at the Center for Gastroenterology and Digestive Endoscopy of Natal and the Onofre Lopes University Hospital (HUOL) of the Federal University of Rio Grande do Norte, located in the city of Natal, Rio Grande do Norte, Brazil, diagnosed with SCP were evaluated. The study was approved through the opinion of the Ethics and Research Committee number: 4.382.180 of November 5, 2020.

Patients were diagnosed with SCP and were therefore included if they had one of the following conditions: (1) typical SCP identified using computed tomography, magnetic resonance imaging (MRI), or endoscopic ultrasound (EUS). The lesion considered typical was microcystic lesion according to the classification of Sahani^22^, meaning a lesion with multiple (usually >6) and small (<2 cm) group cysts. Typically, a lack of communication of the cyst with the pancreatic ducts was necessary for the diagnosis. A central scar, though typical, was not considered necessary for the diagnosis due to its infrequency; (2) nontypical cystic lesions, without communication with pancreatic ducts, identified using EUS in which the dosage of carcinoembryonic antigen was <192 µg/L; and (3) SCP confirmed using anatomopathological testing in cases undergoing surgical resection.

Furthermore, patients who presented the following conditions were excluded to avoid the inclusion of pancreatic pseudocysts: (1) alcohol intake >40 g of ethanol per day for >5 years ; (2) diagnosis of chronic pancreatitis; (3) history of acute pancreatitis prior to the diagnosis of SCP; and (4) history of abdominal trauma.

For the investigation of patients with pancreatic cysts, either computed tomography or MRI was initially requested. Computed tomography was more frequently requested in the first half of the study and then replaced by MRI, which started to be preferentially requested. As EUS is the most invasive exam, it was indicated only in the case of diagnostic doubt. This latest exam became available to patients at the Center for Gastroenterology and Digestive Endoscopy and the HUOL in 2012 and 2017, respectively.

Surgery was indicated in the following conditions: (1) in the presence of typical symptoms related to the injury (acute pancreatitis and symptoms related to compression by the cyst of the adjacent structures) and (2) in diagnostic doubt, meaning when the diagnosis did not meet the first two inclusion criteria for the study. Once the surgery had been indicated, only fit patients were referred to the procedures.

The type of surgery for the patients was determined by the location of the lesion in the pancreas and also by the relationship of the lesion with the main pancreatic duct. As of 2013, laparoscopic access became the procedure of choice for pancreatic body and tail injuries. Silicone tubular or laminar drains (1 or 2) were routinely used. The drains were kept for at least 6 days and removed based on the amylase value from the seventh day onward. The complications of the procedures were categorized according to the Clavien-Dindo classification^14^, with the diagnosis of pancreatic fistula being defined by the International Study Group of Pancreatic Surgery^5^. Thus, the pancreatic fistula was defined when the fluid amylase value in the drains was greater than three times the upper limit for serum amylase^2^ at any time from the third postoperative day. Mortality was considered up to 90 days after surgery. Patients who were treated conservatively underwent clinical and radiological follow-up.

## RESULTS

A total of 97 patients were diagnosed with PCN during the study period. Of these, 27 (27.8%) patients were included in this study because they had SCP. There were 23 (85.18%) women and 4 (14.81%) men, with a mean age of 63.4 (31-89) years. The most prevalent comorbidities were systemic arterial hypertension reported by 37.03% of patients (n=10), followed by diabetes mellitus in 33.33% of patients (n=9). Only one patient, who aged 89 years, had typical symptoms related to SCP with dementia, with a typical SCP imaging exam of 7.0 × 6.1 cm in the head of the pancreas, and with two previous episodes of pancreatitis.

Most patients underwent MRI (62.9%), while computed tomography was the second most requested method (37.0%). EUS was performed in eight patients (29.6%). Of those undergoing EUS, six patients were in the conservative treatment group, and the other two patients were in the group who underwent surgery.

The lesion was single in 88.9% of patients (n=24), while two or more lesions were found in 11.11% of patients (n=3). Typically, the intraductal papillary mucinous neoplasm was the second diagnosis when more than one lesion was found. The mean lesion size was 4 cm (1.2-10 cm). Calcification was identified in 18.5% (5/27) of the cases, being more frequently located in the center of the lesion. The pancreas lesion was located in the body/tail of the pancreas (55.5%) in most cases, while the pancreas head/uncinate process was the second most frequent location (44.4%).

**Figure 1 -:**
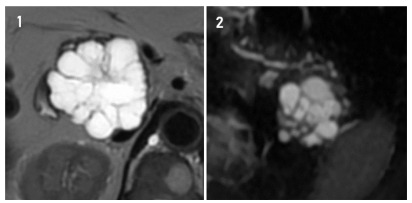
Two patients with typical SCP, both on MRI. 1: A microcystic lesion in the head of the pancreas measuring 5.8 × 5.2 cm is identified. 2: A microcystic lesion in the pancreas tail measuring 4.5 × 4.2 cm is identified.

**Figure 2 -:**
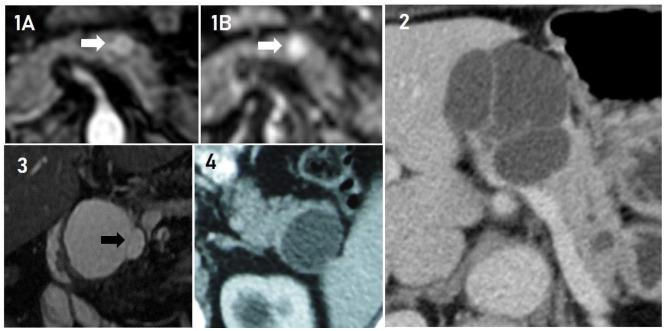
Four patients with atypical SCP. 1A and 1B: SCP solid appearance in MR I. In 1A, the examination of the arterial phase showing a 1.0 cm lesion with arterial uptake in the pancreas body (denoted with white arrow). In 1B, it is observed that the lesion restricted diffusion (denoted with white arrow). 2: Macrocystic lesion in the pancreas body. 3: 5.0 cm unilocular lesion with septum (septum is denoted with black arrow) in uncinate process of pancreas. 4: Unilocular lesion without septa in pancreas tail measuring 3.7 × 3.2 cm.

Regarding morphology, the microcystic lesion was found in 66.6% (18/27) of the cases; unilocular lesion with or without septa was found in 22.2% (6/27) of the cases; macrocystic lesion was found in 7.4% (2/27) of the cases; and a solid lesion was found in 3.7% (1/27) of the cases (Figures 1 and 2). Communication with the main pancreatic duct was suggested in MRI in two cases, one with a microcystic lesion and the other with a unilocular lesion with septum. However, the communication suggested in the MRI was not confirmed in the anatomopathological testing in either of these two cases. The main pancreatic duct was dilated (between 5 and 9 mm) in two cases (7.4%), and this dilation was attributed to compression by the lesion in both cases. The characteristics of the lesions are shown in Table 1 and Figures 1 and 2. The analysis of carcinoembryonic antigen and fluid amylase aspirated from the cyst was performed in four patients. The mean dosage of carcinoembryonic antigen was 198.25 ng/mL (0.2-530). The value was considered typical of SCP in two patients, as it was <192 ng/mL. The mean amylase value was 6.154 U/L (43-12.458 U/L).

**Table 1 -:** Characteristics of cysts and etiologies of surgical indication.

Number of lesions (n=27)	
One	88.9%
>1	11.1%
Size (n=27)	
4 (1.2-10 cm)	
Location (n=27)	
Body/tail	55.5%
Head/uncinate process	44.5%
Morphology (n=27)	
Microcystic	66.6%
Unilocular	22.2%
Macrocystic	7.4%
Solid	3.7%
Pancreatic duct (n=27)	
Dilated 5-9 mm	7.4%
Normal	92.6%
Calcification (n=27)	
Present	18.5%
Absent	81.5%
Surgery indication (n=10)	
Suspected mucinous cystadenoma	70%
Suspicion of IPMN with worrying factors (FUKUOKA)	20%
Suspected neuroendocrine tumor	10%

Surgical treatment was performed in 10 cases (37.0%; 10/27) and corresponded to 34.5% (10/29) of the surgical indication cases for PCN and 7.3% (10/136) of all pancreatectomies performed by the first author (ECA) during the study period. Surgery was performed at baseline in six cases, while it was performed after a mean follow-up period of 26.2 months in four cases (8-54). The indication for surgery was motivated due to a suspicion of mucinous cystadenoma in seven cases (due to unilocular lesions without septa in the pancreas body/tail in four of these cases; due to macrocystic lesions in two cases; and due to unilocular lesion with septum in one case). Surgical indication in two cases was due to a suspicion of intraductal papillary mucinous neoplasia from identifying supposed communication with the main pancreatic duct associated with “worrying factors.” Finally, the surgical indication in one case was due to a solid arterialized lesion and suspicion of neuroendocrine tumor. The surgical procedures indicated were: body-caudal pancreatectomy with splenectomy (60% (6/10) - 4 of which by laparoscopic access); gastroduodenopancreatectomy (20%); enucleation (10%), and central pancreatectomy (10%). The patients did not present any complications, having Clavien-Dindo grade I and Clavien-Dindo grade II in 30%, 40%, and 30% of cases, respectively. Clinically relevant pancreatic fistula (grades B and C) was present in 30% of cases. The average length of stay was 10.1 days (5-29 days), and mortality was nil.

## DISCUSSION

The PCN approach has seen clear progress over the past 30 years, while systematic surgical resection was advocated by most authors^26^ in the 1990s, due to improvements in imaging tests, and also due to better knowledge of the diseases involved; this approach is currently considered unacceptable.

SCP is a frequent PCN and is often considered asymptomatic, with a benign clinical course and associated with negligible potential for malignancy. For these reasons, it is considered one of the best examples in which pancreatectomy should be avoided^17^.

In the largest series of PCNs submitted to resection and, therefore, with diagnostic confirmation, Valsangkar et al.^25^ studied 851 patients at the Massachusetts General Hospital from 1978 to 2011. By comparing the first (1978-1989) with the last period of the study (2005-2011), the authors observed a drop from 26.9% to 11.7% in the prevalence of surgeries for SCP. However, recent series continue to show that a significant proportion of patients still undergo surgery. For example, including 322 cases of PCN undergoing resection, Anonsen et al.^3^ found SCP as the cause of surgery in practically one-fourth (23.9%) of all cases. A greater number of surgeries in patients with SCP were found in a large multicenter study conducted in 71 centers in 23 countries, which involved 2,622 patients with SCP. The rate of pancreatectomy in this study was 60%^17^.

Pancreatectomy continues to be a procedure with a high morbidity rate and, therefore, establishing a correct SCP diagnosis without the need for pancreas resection has been and will increasingly be the objective in managing these patients. Thus, characterizing the morphological aspect of the lesion by modern imaging exams available, particularly MRI, is adequate and sufficient in most cases.

Since its original description by Compagno and Oertel^11^ in 1978, when it was called “microcystic adenoma,” SCP has been typically described as a cystic pancreatic lesion where small (a few millimeters up to <20 mm) and multiple (>6 mm) cysts are grouped in a “bunch of grapes” aspect^22^. When this aspect is found, the diagnosis can be safely performed without the need for further tests. The problem is that for many years, other types of morphological features have been recognized in SCP. About 30 years ago, Lewandrowski et al.^20^ described five cases of “microcystic adenoma” with macrocystic characteristics and suggested at the time that the term “microcystic adenoma” be changed to SCP, which ended up happening. Other atypical forms for SCP, such as unilocular and even solid lesions, have also been described. Such atypical forms of SCP have made its diagnosis difficult^10^.

Sperti et al.^24^ studied seven cases of patients with unilocular SCP. The majority were women with a mean age of 57.1 years and with lesions predominantly in the pancreatic head. The size ranged between 1.3 and 15 cm. The authors concluded that an SCP diagnosis could be suggested by identifying unilocular lesions in patients without a history of pseudocyst and with low levels of tumor markers in the blood and fluid aspirated from the cyst. Likewise, including a series of eight patients with macrocystic SCP, Chatelain et al.^9^ observed women, mean age of 48 years, with a prevalence of asymptomatic lesions and with lesions evenly distributed throughout the pancreas. The mean size of the lesions was 3 cm, being considered smaller than that found in the microcystic SCP. Although most patients underwent surgery for suspected mucinous cystadenoma, similar to the study by Sperti et al.^24^ and our study, millimetric cysts along the main cyst wall were found during EUS in three cases, which led the authors to suspect atypical SCP.

Our study essentially suggests that the atypical morphological finding of SCP is frequent and that this is the main cause for surgical indication in these patients. In analyzing the complete sample, 33.3% of patients (9/27) had atypical lesions. Of these, an oligocystic or macrocystic lesion was found in 88.8% (8/9) of the cases, while the solid form of the SCP was found in 11.1% (1/9) of the cases. By analyzing only the operated cases, we observed that the surgical indication in 90% of the cases was motivated by identifying atypical lesions confused in most cases with mucinous cystadenoma.

In most reviews of PCN, the prevalence of atypical presentation in patients with SCP, particularly oligocystic or macrocystic forms, has been described in 10% or less of patients^8^ ^,^ ^19^ ^,^ ^21^. However, this low prevalence is not a consensus in the literature.

Jais et al.^17^ analyzed 2,622 patients in a study with the largest number of patients with SCP ever involved and found an index of macrocystic lesion and combined macrocystic and microcystic lesions of 32% and 18%, respectively. The typical microcystic appearance was observed in 45% of cases. This disagreement between studies on the prevalence of atypical lesions in SCP suggests that their real incidence is not known. If, on the one hand, a higher rate of atypical findings is to be expected in surgical series, on the other hand, in series with only a presumptive diagnosis without histological confirmation, it is impossible to assume that several other cases of atypical lesions not included were not SCP.

One of the important aspects in the analysis of pancreatic cystic lesions is the relationship of the cyst with the main pancreatic duct. Typically, and unlike intraductal papillary mucinous neoplasm and pseudocyst, SCP does not communicate with the ductal system. Although MRI is the best test for diagnosing this communication, this correlation was not perfect in our study. For example, communication with the pancreatic duct, which did not exist, was suggested by MRI in two cases. This was the second determining factor for indicating surgery in our series and was particularly important in at least one of the cases with a typically microcystic lesion. The finding of supposed communication between the cyst and the main pancreatic duct can mainly happen in large SCP in the pancreas head, in which simple compression of the duct can lead to dilation. Thus, the large cyst close to the dilated duct ends up simulating communication^1^.

Puncture with analysis of the aspirated liquid during EUS has been suggested by some as an important tool to improve the diagnosis in doubtful cases of pancreas cysts^4^ ^,^ ^7^ ^,^ ^12^. Despite this, most guidelines only recommend EUS as an adjunct to computed tomography and, preferably, to MRI^15^ ^,^ ^16^. While molecular analysis (GNAS, KRAS mutation) is scarcely available, the carcinoembryonic antigen and amylase dosage is easily accessible in our environment. In our study, EUS was only performed in eight cases (29.6%), preferably when conventional imaging exams were unreliable. The SCP diagnosis was confirmed by the typical morphological aspect of a microcystic lesion in half of these cases, which was not evident in the previously performed imaging exam. In this situation, the endoscopist chose not to puncture the lesions. However, the puncture and carcinoembryonic antigen and amylase dosage were performed in the other four cases. The carcinoembryonic antigen value was <192 ng/mL in two of these cases, corroborating the diagnosis of nonmucinous lesion and avoiding surgery, while the value was higher in the other two cases, which ended up contributing to the surgical indication for suspected mucinous cystic lesion of the pancreas. Thus, no conclusions can be drawn about the effectiveness of EUS in the differential PCN diagnosis since only a small number of patients were examined. Despite this, the present study suggests a protective effect in avoiding surgery when the patient was undergoing EUS, since EUS was four times more frequent in the conservative treatment group.

Several studies have analyzed different carcinoembryonic antigen values ​​in the aspirated fluid with the aim of differentiating mucinous from nonmucinous lesions. The carcinoembryonic antigen cutoff value of 192 ng/mL used in the present study was derived from an important multicenter study involving 341 patients undergoing pancreatic cyst puncture, which found better accuracy in differentiating between mucinous and nonmucinous cysts at this value^7^. However, there is much controversy in the literature about what would be the best cutoff value to define the nature of these cysts. There appears to be a wide “gray zone” between a low value that is very specific for nonmucinous lesions and a high value that is very specific for mucinous lesions. Lowering or increasing the cutoff to increase specificity greatly reduces sensitivity for both diagnoses^6^.

It is very likely that once new modern endoscopic strategies are validated and made available in clinical practice, such as cyst wall microbiopsy, confocal laser scanning endomicroscopy, and intraductal ultrasonography^18^, they may soon be responsible for better refinement and accuracy of the PCN diagnosis.

Our study has some limitations: (1) the number of patients included is small; (2) it is a series of patients extracted from surgery outpatient clinics and, therefore, it is expected that there will naturally be a bias in the selection of difficult-to-diagnose cases; and (3) EUS was performed in just under 30% of cases.

## CONCLUSION

The currently available alternatives for diagnosing SCP are imperfect. The atypical morphological presentation of SCP is frequent and is mainly responsible for indicating surgery in these cases. More liberal performance of EUS in all nonpseudocyst unilocular cysts in which mucinous cystadenoma is suspected may help to avoid surgeries in atypical SCP. It is crucial that new diagnostic methods should be developed in order to reduce the number of unnecessary pancreatectomies.
